# STED Nanoscopy with Time-Gated Detection: Theoretical and Experimental Aspects

**DOI:** 10.1371/journal.pone.0054421

**Published:** 2013-01-18

**Authors:** Giuseppe Vicidomini, Andreas Schönle, Haisen Ta, Kyu Young Han, Gael Moneron, Christian Eggeling, Stefan W. Hell

**Affiliations:** 1 Nanophysics, Istituto Italiano di Tecnologia, Genoa, Italy; 2 Department of NanoBiophotonics, Max Planck Institute for Biophysical Chemistry, Göttingen, Germany; 3 Department of Neuroscience, Institut Pasteur, CNRS URA 2182, Paris, France; 4 Weatherall Institute of Molecular Medicine, University of Oxford, Oxford, United Kingdom; University of Milano-Bicocca, Italy

## Abstract

In a stimulated emission depletion (STED) microscope the region in which fluorescence markers can emit spontaneously shrinks with continued STED beam action after a singular excitation event. This fact has been recently used to substantially improve the effective spatial resolution in STED nanoscopy using time-gated detection, pulsed excitation and continuous wave (CW) STED beams. We present a theoretical framework and experimental data that characterize the time evolution of the effective point-spread-function of a STED microscope and illustrate the physical basis, the benefits, and the limitations of time-gated detection both for CW and pulsed STED lasers. While gating hardly improves the effective resolution in the all-pulsed modality, in the CW-STED modality gating strongly suppresses low spatial frequencies in the image. Gated CW-STED nanoscopy is in essence limited (only) by the reduction of the signal that is associated with gating. Time-gated detection also reduces/suppresses the influence of local variations of the fluorescence lifetime on STED microscopy resolution.

## Introduction

Far-field fluorescence microscopy is a powerful imaging tool for investigating (living) cells due to its non-invasive access to the cellular interior, the specific and sensitive detection of cellular features through fluorescence tagging, and the simple sample preparation. However, many features are too small to be discerned with standard light microscopy, whose spatial resolution is curtailed by diffraction to 200–350 nm [1].

Stimulated emission depletion (STED) microscopy [2], [3] overcame the diffraction barrier and increased the spatial resolution of fluorescence microscopy for the first time by a large factor; in principle it can reach resolution at the molecular scale. For this purpose, STED microscopy (or nanoscopy) uses stimulated emission to inhibit fluorescence emission at predefined sample coordinates such that adjacent features emit sequentially in time. Similarly, other fluorescence inhibition processes may be used to overcome the diffraction barrier [4], such as the shelving into metastable dark states in ground-state depletion (GSD) nanoscopy [5], [6] or the use of photoswitchable fluorescence markers in the generalized concept called RESOLFT [4], [7], [8]. The strategy of modulating the fluorescence emission of neighbouring features has also been exploited in more recent far-field fluorescence nanoscopy approaches [9], [10], [11], [12], [13] that perform on-off fluorescence switching molecule by molecule randomly in space. Meanwhile, STED nanoscopy has addressed many questions in biology [14], [15], [16], [17] and its implementation has become simple [18], [19], [20]. STED currently also provides the fastest subdiffraction resolution recordings [21].

In a typical STED microscopy implementation, a laser beam inducing stimulated emission and featuring at least one zero-intensity point is overlaid with a regularly focused excitation beam. Thus, the STED beam inhibits fluorescence emission everywhere but at the zero-intensity points. A common design is a doughnut-shaped focal intensity pattern of the STED beam. If the intensity of the STED beam at the doughnut crest *I*_STED_ strongly exceeds the value *I*_s_ at which half the fluorescence is suppressed, the effective fluorescence signal is confined to subdiffraction dimensions. Scanning the co-aligned excitation and STED beams through the sample yields the final subdiffraction resolution image, whereby the resolution can be adjusted by the intensity of the STED beam.

STED nanoscopy can be implemented with both continuous wave (CW) [18], [22] and pulsed lasers [23]. The latter modality relies on synchronized trains of excitation and STED pulses with the pulses of the STED beam reaching the focal plane simultaneously or right after the excitation pulses, but within a fraction of the lifetime of the fluorescent state [23]. Pulses of the order of 0.1–1 ns suppress undesired polarization effects [24], [25], jitter in pulse timing [26], multi-photon excitation [27], and photo-bleaching [28].

Although CW STED beams simplify the implementation of STED nanoscopy, the less efficient spatial confinement of fluorescence associated with this method is disadvantageous. Unlike in the pulsed mode, where all the photons (of the STED pulse) act shortly after the excitation event, in the CW implementation, the instantaneous STED intensity is typically lower, and so is the instantaneous probability, i.e. the rate, of stimulated de-excitation. A non-negligible part of the molecules still emits fluorescence because they have not been exposed to enough de-exciting photons. Such fluorescence is particularly prevalent right at the slopes of the zero-intensity point of the STED beam where the STED beam is weaker thus contributing to blur [26]. In other words, the suppression of fluorescence strongly depends on the number of STED photons to which the molecule is exposed while residing in the excited state.

It has been known that in a pulsed STED scheme the fluorescence photons should be detected right after the STED pulse has left [24], [29]. This has also been shown in an experiment combining time-correlated-single-photon-counting and pulsed STED nanoscopy [30]. Likewise, the generalization of the STED principles to other optical transitions between two distinct states has shown that for constantly acting (CW) beams, the obtainable effective resolution scales with the duration of the action of the beam [4]. Thus, by applying pulsed excitation and time-gated detection, the residual fluorescence produced at the slopes of the CW STED beam can be solved by detecting fluorescence only from molecules that have been exposed to the beam for a duration>*T*_g_ after excitation [31], [32]. In fact, recent experiments have established gated CW-STED microscopy as a simple but powerful approach to observe the cellular nanoscale, including of living cells [32]. However, despite its recent popularity many physical aspects of this recording mode, as well as its benefits and limitations have not been elucidated.

In this paper, we therefore develop a theoretical framework describing the time evolution of the effective point-spread-function (PSF) of a STED nanoscope, thus quantifying the resolution obtained by time-gated detection. Together with experimental data, this framework provides a comprehensive view of the performance of gated STED nanoscopy in both the all-pulsed (P-STED) and the CW-STED (pulsed excitation, CW STED) modalities, especially with respect to the choice of the time-gated detection window and the reduction of the signal-to-noise or -background ratio. While hardly any improvement is expected for time-gated P-STED, the time-gated detection not only increases the effective spatial resolution of CW-STED nanoscopy but also reduces adverse effects of fluorescence lifetime heterogeneities in the sample.

## Materials and Methods

### Sample Preparation

We used ∼40 nm large fluorescent beads (Crimson beads, Invitrogen, Carlsbad, CA; excitation and emission maxima at 625 nm and 645 nm, respectively) and ∼35 nm large fluorescent nano-diamonds (FNDs) [33] (Institute of Atomic and Molecular Sciences, Academia Sinica, Taipei, Taiwan; excitation and emission maxima at 560 nm and 700 nm, respectively) for the experimental characterization of the effective point-spread-function (PSF) of our STED nanoscope. A dilute dispersion of the fluorescent beads was prepared by drop casting a solution of the beads on a poly-L-lysine (Sigma, Saint Louis, MO) coated glass coverslip and mounting it with Mowiol (Sigma-Aldrich, Taufkirchen, Germany). The FND sample was prepared by spin-coating the particles in poly(vinyl-alcohol) (PVA) on a microscope cover glass.

The mammalian PtK2 cell line was grown as described previously [34]. Cells were seeded on standard glass coverslips to a confluence of about 80% and fixed with ice-cold methanol (−20°C) for 4 min followed by an incubation in blocking buffer (PBS containing 1% BSA). Microtubules were stained using an immunofluorescence labelling protocol [35] involving a primary antibody (anti β-tubulin mouse IgG (monoclonal), Sigma) and a secondary antibody (sheep anti-mouse IgG, Dianova, Hamburg, Germany) labelled with the organic dye ATTO647N (Atto-Tec, Siegen, Germany) or KK114 [36]. All antibodies were diluted in blocking buffer and incubated for 1 h each followed by several washing in blocking buffer. Mounting was again performed with Mowiol.

### STED Nanoscope

Our STED nanoscope setup [32] featured a 532 nm (PicoTA, PicoQuant, Berlin, Germany) and a 635 nm (LDH-D-C-635, PicoQuant) pulsed diode laser for excitation and a Ti:Sapphire laser (Mira900, Coherent, Santa Clara, CA) for STED, which was tuned to 740 or 760 nm and operating either in the CW or in the mode-locked pulsed mode with a repetition rate of 76 MHz. The STED light was guided through two glass rods and coupled into a 120 m long polarization maintaining single mode fiber (AMS Technology, München, Germany), which in the pulsed modality stretched the pulse width to ∼250 ps. In the pulsed STED modality, the excitation diode lasers were synchronized to the STED laser by a home-built electronic delay unit. In the CW modality, the repetition of the pulsed excitation lasers was tuned to 40 or 80 MHz, based on the application. The doughnut-like intensity distribution of the STED light was created by introducing a polymeric phase plate (RPC Photonics, Rochester, NY) applying a helical phase ramp of exp(*iφ*), with 0<*φ*<2*π* in the STED beam that was then imaged into the back aperture of a 1.4 NA objective lens (HCX PL APO, 100×/1.40, oil, Leica, Wetzlar, Germany). Excitation and STED beams were aligned on the same optical axis using custom-made dichroic mirrors (AHF Analysentechnik, Tübingen, Germany). The fluorescence was detected through the same objective lens, filtered out with appropriate bandpass filters to reject laser scattering and imaged onto a multimode optical fibre with an opening of the size of about an Airy disc of the imaged excitation PSF. The fibre was attached to a single-photon-counting module (id100-MMF50, id Quantique, Carouge, Switzerland) and connected to a time-correlated single-photon-counting board (SPC-730, Becker & Hickl GmbH, Berlin, Germany). The image acquisition was performed by scanning the sample with a 3D piezo stage (NanoMax TS 3-axis, Thorlabs GmbH Europe, Dachau, Germany). The STED and confocal reference images were recorded simultaneously on a line-by-line basis by opening and closing a mechanical shutter in the STED beam.

### Intensity and Power Measurements

Both for the STED and the excitation light we indicate the average power *P* measured at the back aperture of the objective. Due to losses in the objective, the power at the sample is actually lower by 30% and 25% at 760 nm and 740 nm, respectively. The average STED intensity at the doughnut crest can be estimated by *I_STED_* = *kP_STED/_A_STED_* with *A_STED_* denoting the STED focal area of a nearly diffraction-limited light spot; *k = *0.3 is a scaling factor correcting for the doughnut-shaped intensity distribution. We determined *A_STED_* ≈π(FWHM_STED_/2)^2^ from the diameter FWHM_STED_ of a regularly focused (nearly Gaussian) spot. The value of FWHM_STED_ ≈350 nm was measured from a scattering gold bead of sub-diffraction diameter (80 nm gold colloid, En.GC80, BBinternational, Cardiff, UK) in a non-confocal mode. For the P-STED modality the transient (or peak) intensity during a rectangular pulse of duration *T*_STED_ is given by *I*_STED_^*^ *= I*_STED_/(*fT*_STED_) with *f* being the repetition rate of the laser.

### Lifetime and Signal-to-noise Ratio Analysis

We performed fluorescence lifetime recordings and analysis using time-correlated single-photon-counting (TCSPC) and a maximum-likelihood estimation method with a Poissonian assumption of the error distribution [37]. The fitting to the experimental TCSPC data included a multi-exponential decay with *i* components, ∑_i_ *α*_i_exp(−*t*/*τ*_i_), and a convolution with the instrument response function. For each of the *i* components *τ*_i_ represents the decay time; *α*_i_ is a photon-weighted amplitude. Consequently, each component *i* contributes a fraction *c*_i_ = *α*_i_τ_i_/∑_j_(α_j_τ_j_) to the fluorescence signal and the mean decay time (intensity-weighted average lifetime) is given by <τ> = ∑_i_ *c*_i_ *τ*_i_. The instrument response function was measured on a purely scattering sample.

To quantify the signal-to-noise ratio (SNR) and signal-to-background ratio (SBR) of the experimental images, we defined the peak SNR (PSNR) and peak SBR (PSBR). The PSNR and PSBR represent the SNR and SBR in the brightest part of the recorded images. With *g*(*i*) giving the photon count rate recorded (gated or un-gated) at the pixel *i* of an image (i.e. the number of counts detected per pixel dwell-time at pixel *i*)
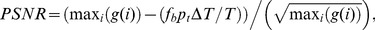
(1)where *f*_b_ is the uncorrelated background count rate, *p*_t_ the pixel dwell-time, and *ΔT/T* = (*T*−*T_g_*)/*T* the time-gated fraction of the pulse period *T*. *f*_b_ was directly estimated from the late time-bins of the TCSPC histogram (the histogram of the photon arrival times): when a photon has been registered in a late time-bin it has been most likely generated by an uncorrelated background source. Following the same notation we defined the PSBR as
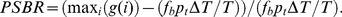
(2)


### Theory

The main equations governing the theory of time-gated detection for STED nanoscopy have been reported [31], [32]. Starting from the temporal evolution of the fluorescence signal under stimulated emission, the spread of coordinates where fluorescence is allowed and hence registered, i.e. the effective point-spread-function (E-PSF) of a time-gated STED nanoscope, has been derived. However, the gain in resolution in a gated STED nanoscope over time has not been explicitly quantified yet. To this end, we analyze the characteristics of the E-PSF as a function of the time of action of the CW-STED beam. We shall denote this time-dependent E-PSF as tE-PSF throughout the manuscript.

### Fluorescence Signal Under Stimulated Emission

At first we derive the temporal dynamics of the fluorescence signal under stimulated emission. We make several assumptions: (i) The fluorescent marker is described by a simple two-level model consisting of a ground *S*_0_ and a first excited electronic state *S*_1_; dark states and vibrational sub-states are neglected. (ii) Excitation from *S*_0_ to *S*_1_ by the STED light is neglected as well. (iii) The fluorophores are initially in their *S*_1_ state due to a brief excitation pulse. We also assume that the time period *T_ = _*1/*f* between two pulses is longer than the excited-state lifetime *τ* of the markers, i.e., all markers have relaxed to *S*_0_ before the arrival of the next excitation pulse; hence, the conditions at the beginning of every excitation cycle are the same. (iv) Spontaneous *S*_1_ → *S*_0_ de-excitation takes place with a rate constant *k*_S1_ *= 1/τ*, (with *τ* denoting the excited state lifetime), and fluorescence photons are emitted with a quantum yield *q*_fl_, i.e., with a rate *k*_fl_ = *k*_S1_ *q*_fl_. (v) We evolve our calculations from the pulsed STED modality assuming rectangular STED pulses with a temporal width *T*_STED_, and generalize for the CW-STED case by setting *T = T_STED_*. (vi) The rate of stimulated emission during the pulse is given by *k*_STED_ = σ_STED_^∼^ *I*_STED_^*^ with σ_STED_^∼^ = *σ*_STED_ *λ*_STED_ */*(*hc*) being the stimulated emission cross section divided by the photon energy (*λ*_STED_ the wavelength of the STED light, *hc* = 1.99·10^−25^ Jm is the product of Planck’s constant and the velocity of light) and *I*_STED_^*^ = *I*_STED_ *T/T_STED_* the transient STED intensity. *I*_STED_ defines the time-averaged STED intensity derived from the directly measurable average power of the beam. (vii) We define a transient saturation intensity I_s_^*^ as the transient STED intensity at which *k*_S1_ = *k*_STED,_ i.e. I_s_^*^ = k_S1_/σ_STED_^∼^, revealing a transient suppression or saturation factor ς^*^ = *I*_STED_^*^/I_s_^*^ = k_STED/_ *k*_S1_. (viii) STED experiments usually apply a circular polarization of the STED light; therefore, we neglect orientation or rotation characteristics of the fluorophore, which can decrease the efficiency of stimulated emission [24], [25].

The fluorescence signal is proportional to the relative population *P*_S1_ of the first excited state *S*_1_, whose change over time *t* can be expressed by the rate equation
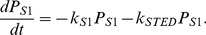
(3)With *P*_S1_(0) = 1, the fluorescence emission rate at a time *t* after excitation is



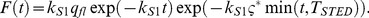
(4)The first and second exponentials describe the spontaneous decay and the action of the STED beam, respectively.

### Time Evolution of the E-PSF (tE-PSF)

In previous theoretical work on STED, the fluorescence signal was usually integrated over time and the temporal evolution discarded. Here we regard the temporal evolution of the fluorescence signal under stimulated emission, calculating the tE-PSF. At low excitation intensities (no saturation of the excited state) the spatial modulation of the probability to excite a fluorophore follows the excitation intensity profile. Hence, the tE-PSF of a STED microscope *h*(*t*,*r*) can be derived by the product of the excitation intensity profile *h*_exc_(*r*), the probability of fluorescence emission *F*(ς^*^(*r*),*t*) given in Equation (4), and the detection efficiency profile *h*_det_(*r*). For an analytical description of the tE-PSF we approximate the product *h*_c_ *= h*_exc_ *h*_det_ by a Gaussian distribution with a full-width-at-half-maximum (FWHM) *d*_c_ and an amplitude equalling unity, i.e., *h*_c_(*r*) = exp(−4ln2 *r* ^2^/*d*_c_^2^). In the vicinity of the zero-intensity point (*r* = 0), the STED intensity profile of the doughnut can be approximated by a parabola *I_STED_*(*r*) ≈4*I*_STED_ *a* ^2^ *r* ^2^, with the intensity *I*_STED_ at the doughnut crest, and a constant *a* that depends on the shape of the doughnut minimum [38]. Using Equation (4), the tE-PSF for 0≤t≤ *T_STED_* reads
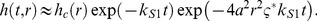
(5)


**Figure 1:**
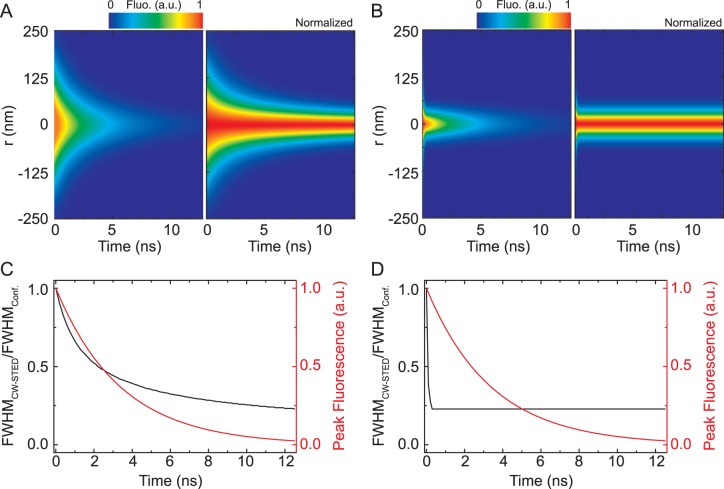
Calculated time evolution of the observation volume of STED nanoscopy (tE-PSF). Lateral (*r*) intensity distribution *h*(*t*,*r*) (A,B), the full-width-at-half-maximum (FWHM(*t*)) (C,D; black, left axis) and the peak signal *h*(*t*,0) (C,D; red, right axis) of the tE-PSF as a function of the time of the STED beam action (excitation at time 0) for the CW-STED (A,C) and P-STED (B,D) modality (A,B; left panel: original, un-normalized data; right panel: normalization to 1 for each time). The excitation intensity profile *h*_exc_(*r*), the detection efficiency profile *h*_em_(*r*) and the STED intensity profile *I*_sted_(*r*) are computed using Fourier theory [39]. Given *I*_sted_(*r*), *h*_exc_(*r*) and *h*_em_(*r*) the time evolution of the observation volume *h*(*t*,*r*) is calculated using Equation (4). We assumed an oil immersion objective of 1.4 numerical aperture, *τ*_ = _3.4 ns, *T* = 1/80 MHz, *T*_STED_ = 300 ps, *λ*(excitation)_ = _635 nm, *λ*(STED)_ = _760 nm and *λ*(emission)_ = _670 nm, the same average powers for both modalities, i.e. ς^*^ = 4.8 and 200 for CW and pulsed mode, respectively, and a detection pinhole with a projected diameter of 500 nm in the sample space (0.9 × the Airy disc diameter).



For *T_STED_* ≤t≤*T* we have
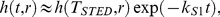
(6)i.e. the spatial shape (*r*-dependence) of the tE-PSF does no longer change after the STED pulse. We normalized the tE-PSF to unity in the focal centre (*r* = 0) at *t = *0. Examples of the simulated tE-PSF *h*(*t*,*r*) for P- and CW-STED are shown in Figure 1. The tE-PSF is sharpened over time of the STED action accompanied by a decrease of the amplitude, accounting for the spontaneous decay (Equations (5) and (6)). Following Equations (5) and (6), the tE-PSF *h*(*t*,*r*) can be approximated by a Gaussian with amplitude

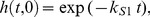
(7)and a time-dependent FWHM
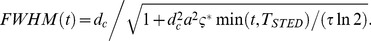
(8)


### E-PSF for Gated Detection

Following the time evolution of the tE-PSF *h*(*t*,*r*), the observation/detection spatial range is reduced by detecting fluorescence at a later point of time *t*, i.e., by performing a time-gated detection. Here, fluorescence is detected only after a time *t* = *T*_g_ from the excitation pulse.

For the P-STED (i.e. all-pulsed) modality with time-gated detection (gP-STED) one usually chooses *T*_g_ ≥ *T*_STED_. In this case, the E-PSF is given by
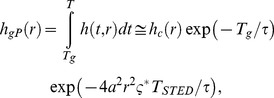
(9)where for the right-hand side we have assumed *T*>>*τ*, i.e. exp(-*T*/*τ*) negligible. By simple computations we obtain
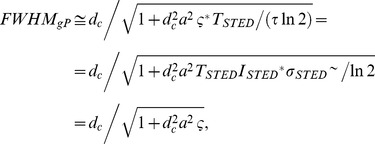
(10)where ς = ς*TSTED/(τln2) = (TSTED ISTED*)σSTED∼/ln2 is the saturation factor for P-STED. It is usually defined as ς = ISTED/Is with the saturation intensity Is being the average intensity at which half the recorded spontaneous emission is suppressed [38]. Obviously Is and thus ς depend on the spectroscopic properties of the molecules and the illumination timing. It is however important to note that ς and thus the FWHM in gated P-STED depends only on the pulse energy (TSTED ISTED*) and the cross section, not on the fluorescence lifetime. We further note that in the absence of a time-gate or in the unusual case of setting Tg<TSTED, i.e. for gate delays shorter than the pulse width of the STED laser, the expression becomes more complex. However, for lifetimes τ>>TSTED (which is usually the case) the integral across the pulse duration, Tg<t≤TSTED, can be neglected in Equation (9) and, in good approximation, Equation (10) remains valid.

This means that under the assumption that the pulse duration is short with respect to the fluorescence lifetime, also for classical P-STED the FWHM does not effectively depends on the lifetime of the fluorophore.

**Figure 2:**
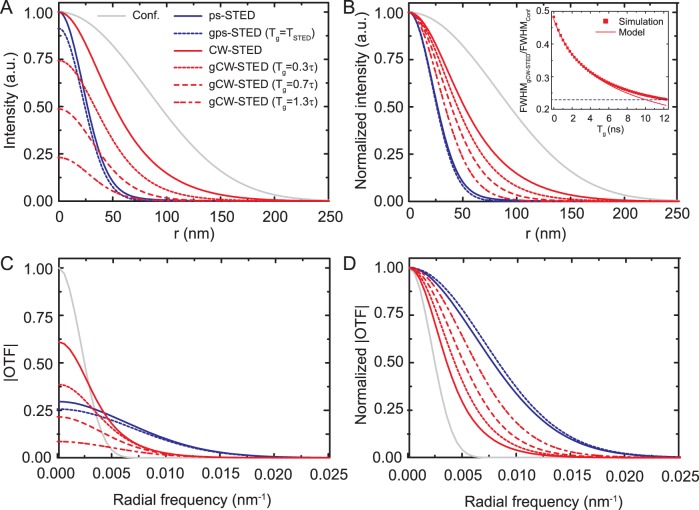
Observation volume under gated detection (E-PSF). Calculated lateral line profiles of the E-PSF (A,B) and OTF (C,D) for confocal (grey line), pulsed STED (P-STED: solid blue line without gating, gP-STED: dotted blue line with *T*_g_ = *T*_STED_) and CW-STED (CW-STED: solid red line without gating, gCW-STED: dotted red lines with different *T*_g_ as denoted) recordings (left panels un-normalized, right panels normalized data). The excitation intensity profile *h*_exc_(*r*), the detection efficiency profile *h*_em_(*r*) and the STED intensity profile *I*_sted_(*r*) are computed using Fourier theory [39]. Given *I*_sted_(*r*), *h*_exc_(*r*) and *h*_em_(*r*) the E-PSF *h*(*r*) is calculated using the time integration of Equation (4). Same parameters as Figure 1. Inset (B): FWHM of the E-PSF of the gCW-STED nanoscope as a function of the time-delay *T*_g_; Red dots depict the simulation values (see above); Red solid line depict the model values: Calculation are based on Equation (12) with *d*_c = _232 nm, *a* = 3.63·10^−3^ nm^−1^, τ = 3.4 ns and ς^*^ = 4.8. The dotted horizontal line represents the FWHM of the simulated E-PSF of the gP-STED nanoscope with *T*_g_ = *T*_STED_ and the same parameters as Figure 1.

In the case of CW-STED with pulsed excitation and time-gated detection (gSTED) the E-PSF *h*_gCW_(*r*) is given by
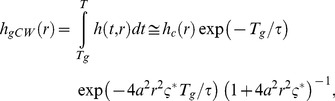
(11)where we have assumed *T*>>*τ*, i.e. exp(-*T*/*τ*) negligible. In the absence of gating (*T*_g_ = 0), the E-PSF has the Lorentzian shape known for the original CW-STED implementation featuring CW excitation [26]. When a gated detection scheme is introduced (*T*_g_>0), the E-PSF consists of an Gaussian term (due to the suppression by the STED light before the detection) and a Lorentzian term (because the remaining excited molecules are then imaged under the same condition of the original CW-STED implementation). A good approximation for the FWHM of Equation (11) is found by replacing the Lorentzian term in Equation (11) with a Gaussian term with the same FWHM (see Supplementary Text S1). We then have



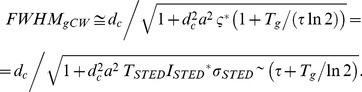
(12)Note that *FWHM*_gCW_(*T*_g = _0) is the FWHM of the Lorentzian E-PSF of the original CW-STED implementation. Unlike for the well-implemented gP-STED, the FWHM of the gCW-STED implementation depends on the fluorescence lifetime *τ*. Albeit the approximation made when deriving Equation (12) values of *FWHM*(*T_g_*) calculated using Equation (12) are similar to those obtained from a rigorous model, which calculates the intensity profiles of the excitation and STED intensities based on Fourier diffraction theory [39] (inset Figure 2B).

A sharpening of the observation/detection area in the sample is observed with increasing time delay *T*_g_ of the time-gated detection, concomitant with a decrease in signal (or amplitude). Notably, the reduction of the FWHM is accompanied by a strong reduction of the pedestal (or Lorentzian tail) of the E-PSF. We note that for very large *T*_g_, Equation (12) slightly underestimates the FWHM of the E-PSF (inset Figure 2B), since for *τ*_ = _3.4 ns and *T* = 1/80 MHz the fluorophores still have a non- negligible probability to be in the excited state before the next excitation pulse arrives, i.e. *h*(*T*,*0*) is not completely zero as assumed.

The E-PSF of the time-gated detection can also be regarded as a weighted sum of different Gaussian distributions with decreasing FWHM and decreasing weights represented by the tE-PSF. Collecting the photons after a time delay *T*_g_ from the excitation pulse, i.e., performing time-gated detection, removes the early tE-PSFs characterized by a larger FWHM and therefore improves the effective resolution at the expense of a loss in overall signal, as outlined in Figures 1 and 2.

For the pulsed STED implementation, our theoretical framework (Figure 2) reveals that collecting the photons immediately after the STED action (*T*_g_ = *T*_STED_) produces the sharpest E-PSF, as expected. The use of a time-delay *T*_g_ larger than the STED pulse width *T*_STED_ only reduces the brightness without further reducing the FWHM of the E-PSF, of course. Furthermore, if the pulse width of the STED beam *T*_STED_ is short compared to the excited-state lifetime *τ* (*T*_STED_/*τ*<<1), which is usually the case, the impact of time-gating is negligible. In our calculations, we have assumed the same average intensity at the doughnut crest *I_STED_* for the P-STED and the CW-STED implementation. The much larger transient intensity *I_STED_* ^*^ = *I_STED_ T*/*T_STED_* of the P-STED modality results in much lower transient saturation factors, ς^*^ = 4.8 for the CW-STED compared to ς^*^ = 200 for the P-STED recordings, and thus by default to a much more confined E-PSF for the P-STED implementation. However, increasing the time delay *T*_g_ of the gCW-STED recordings results in a convergence of the two E-PSFs (Figure 2B). This is not surprising, since the gCW-STED implementation can be viewed as a pulsed implementation whereby the virtual pulse is the exposure time during the gate.

### Optical Transfer Function (OTF)

The optical transfer function (OTF) is the Fourier-transform of the E-PSF in space, meaning that large spatial frequencies (above the noise level) yield features with high spatial resolution. Figures 2C, D compare the OTFs and thus the spatial frequencies transmitted by the imaging modality for different time gates *T*_g_. The increase in effective spatial resolution is obviously not realized by elevating the transmission of large frequencies *per se*. Rather, the transmission of lower frequencies is damped by the gating process, thereby increasing the relative contributions of the larger frequencies. We therefore refer to the improvement in image contrast by an increase in ‘effective resolution’. Similarly to the E-PSF, the OTF can also be regarded as a weighted sum of different OTFs with increasing bandwidth but decreasing strengths, represented by a temporal OTF (tOTF – which are the spatial Fourier-transforms of the respective tE-PSFs). Introducing the gated detection removes the contributions of the early tOTFs, which are mainly characterized by low spatial frequencies.

### Signal-to-noise (SNR) and –Background (SBR) Ratio

As highlighted in Figures 1 and 2, the signal amplitude *h*(0,*t*) of the non-normalized t-EPSF degrades with time. In terms of the OTF, the frequencies boosted by the gated detection might thus be masked by noise (Figure 2C, D). Following our previous considerations a time-delay *T*_g_ decreases the fluorescence signal at the peak of the E-PSF, *h*_gCW/gP_(0), by a factor exp(−*T*_g_/τ), while any uncorrelated background signal is just reduced in proportion to the width Δ*T* = (*T*−*T*_g_) of the detection gate. We defined uncorrelated background as background that is uncorrelated with the pulsed excitation. Important sources of such background are ambient light, fluorescence excited by the STED laser (Anti-Stokes excitation, AStEx) [40] and scattering from the STED beam. Following this definition, background from the excitation light or AStEx background in P-STED [40] is not uncorrelated.

Because all of our images are recorded with a photon counting module, we can assume shot noise as the major source of noise. The shot noise of fluorescence detection scales with the square root of the detected signal. Consequently, the signal-to-noise ratio (SNR) decreases with *T*_g_ as
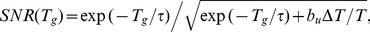
(13)where *b*_u_ is the relative signal level of the uncorrelated background without time-gating. Obviously, the SNR decreases strongly for large gates *T*_g_>>*τ*, simply because all fluorophores will have decayed by then.

The proposed gated detection approach can be also limited by the reduction of signal-to-background ratio (SBR) and thus by the reduction of contrast. In case of uncorrelated background due to the aforementioned ambient light, STED beam scattering and AStEx fluorescence we define a signal-to-background ratio (SBR), which decreases with *T*_g_ as
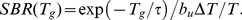
(14)


As for the SNR, the SBR degrades for increasing *T*_g_ and hence the improvement of the effective resolution has to be pondered against the reduced SBR and SNR.

Uncorrelated background signal can certainly be reduced; for example, we have recently presented a digital lock-in method to remove the AStEx background [40]. This method is readily introduced in g-STED nanoscopy as well. Furthermore, for experiments applying low repetition rates, T>>τ, where late detection will be dominated by the uncorrelated background, one may improve the SNR and SBR by detecting only until a time *T*_end_<*T* before the next pulse, i.e., by reducing the detection window Δ*T* = *T*_end_−*T*_g_.

On the other hand, gating significantly reduces correlated background contributions due to scattering of the pulsed excitation or pulsed STED light and due to unspecific background fluorescence of very short lifetime, since these contributions only appear for short times *t*. For example, gated detection has often been applied to increase the SNR/SBR of single-molecule detection experiments [41]. Therefore, it is usually helpful to at least set a time gate *T*_g_ at the end of the excitation pulse.

## Results

### gCW-STED Imaging: Comparison to Theory

**Figure 3:**
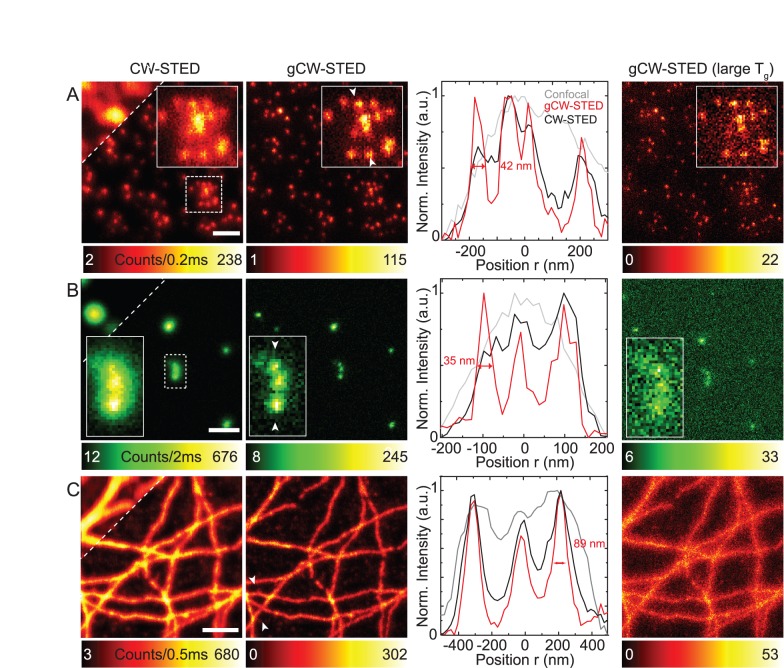
Scanning gCW-STED nanoscopy images. Images of (A) ∼ 40-nm large fluorescent crimson beads, (b) ∼ 35-nm large FNDs, and (c) ATTO647N-immunostained microtubules of a fixed PtK2 cell. The insets (A,B) show a magnified view of the areas marked in the left images. (Left) CW-STED and confocal images (upper left corner), (middle left) gCW-STED images with optimized gating *T_g_* = 1.5 ns, PSNR ∼10 (A), 6 ns, PSNR ∼ 15, PSBR ∼ 26 (B), 1.5 ns, PSNR ∼ 16, PSBR ∼ 13(C), (middle right) normalized intensity profiles along the arrows marked in the insets, and (right) gCW-STED images with relative large *T_g_* = 6 ns, PSNR ∼ 4 (A), 18 ns, PSNR ∼ 5, PSBR ∼ 8 (B), 6 ns, PSNR ∼ 5, PSBR ∼ 3 (C). Excitation: (A) 635 nm, *f* = 80 Mhz, 7 µW; (B) 535 nm, *f* = 40 Mhz, 100 µW; (C) 635 nm, *f* = 40 Mhz, 5 µW. STED: (A) 740 nm, 230 mW; (B) 740 nm, 250 mW; (C) 760 nm 300 mW. Pixel size: (A, B) 10 nm, (C) 20 nm; Uncorrelated background: *f*_b = _20 Hz of dark count rate (A), *f*_b_ = 6 KHz of scattering from the STED beam (B), *f*_b_ = 50 KHz of direct excitation from the STED beam (C); Scale bar: 500 nm (a,b), 1 µm (C).

Our experimental data fully confirms the theoretical considerations regarding the tE-PSF. We first imaged densely packed ∼40 nm large fluorescent crimson beads and ∼35 nm sized fluorescent nano-diamonds (FNDs) to experimentally demonstrate the characteristics of time-gated detection for CW-STED (Figure 3A, B). As expected, a clear rise in effective resolution is obtained by adding the STED light, which is further improved by introducing the gated detection, all in all allowing a much clearer separation of adjacent features. The gCW-STED images of the point-like objects (FNDs and beads) reveal their nominal size (35–40 nm), which indicates that we have reached a spatial resolution of 35 nm or below. These image conditions have been reached at comparatively low average CW-STED powers of *P_STED_*_ = _250 mW for the FNDs and 230 mW for the crimson beads. Note that similar improvements in image resolution on the crimson beads have been obtained previously by the use of a similar STED laser system running in pulsed mode (*T* = 1/80 MHz and *T*_STED_ ≈300 ps) with an average power *P_STED_*_ = _50 mW, i.e., with ∼10 times higher peak STED intensity I_STED_^*^ [38].

Notably, the effective resolution of the gCW-STED images continuously improves with increasing time delay *T_g_* (Supplementary Movie S1, Supplementary Movie S2) up to the limit imposed by the degradation of the SNR and SBR (Equations (13) and (14)).

The fluorescent beads are very bright objects and the uncorrelated backgrounds, including the dark counts background of the detector (few Hz), are negligible in their images. Thereby for increasing time delay the SBR reduction does not represent a substantial problem. The major source of degradation introduce by time gating is the increase of photon counting noise and consequently by optimizing the pixel dwell-time, the images start degrading for relative long time delay *T*_g_>6 ns, compared to the lifetime *τ* ∼ 3 ns of the fluorescent beads (Figure 3A and Supplementary Figure S1).

In contrast the FNDs are less bright objects, and these samples show a relative high level of uncorrelated background due to the scattering of the STED beam (6 KHz). Consequently, even if the SNR of the CW-STED image is high (*T*_g_ = 0, PSNR ∼ 25), the SBR reduces faster and the images degrade already significantly for time delays *T*_g_>18 ns, that are small relative to the FND’s lifetime *τ* ∼ 15–20 ns (Figure 3B). Since caused by scattering, increasing the pixel-dwell time will not improve the SBR.

We next checked our theoretical considerations on the performance of the gCW-STED nanoscope for the imaging of biological samples. Figure 3C shows a gCW-STED image of the microtubule network of fixed mammalian PtK2 cells immunostained with the organic dye ATTO647N. Structural details of this network could much better be visualized in the gCW-STED than in the CW-STED or confocal images. Supplementary Movie S3 shows the evolution of the effective resolution with increasing time-delay *T_g_*. A substantial improvement in effective resolution is already obtained at rather low values *T_g_*<1.5 ns. The quality of the gCW-STED images, however, degrades for relative short time-delays *T*_g_>3 ns compared to the label’s lifetime τ ∼ 3 ns (Supplementary Figure S2). This is mainly due to the uncorrelated background induced by the STED beam (AStEx), which for large *T_g_* dominates over the desired signal.

Importantly, the SBR reduction due to the AStEx or scattering signal induced by the STED laser can be compensated for by using can be compensated using a lock-in detection method able to subtract such uncorrelated background signals [40]. However, one has to keep in mind that also the lock-in system is limited by noise [40].

### gCW-STED Imaging: Lifetime Dependence

In the usual gCW-STED image recording scheme, the STED intensity *I_STED_* is fixed and (apart from strong local optical bias due to polarization [42] or aberration effects) the optical parameters, namely, *d*_c_ and *a*, do not change during the recording. Assuming a constant cross section of stimulated emission *σ*_STED_^∼^ of the fluorescence label, the effective resolution, i.e. the FWHM of the observation volume scales as 1/√(1+ *T_g_*/(τln2)), i.e., with the ratio *T_g_*/τ (Equation (12)). Therefore, the time delay *T_g_* of the gated detection has to be adapted for fluorophores with different fluorescence lifetimes to reach similar effective resolution. For example, the time-correlated single-photon counting (TCSPC) data recorded for the single fluorescent beads showed a single exponential decay with an average lifetime *τ* ∼ 3 ns, while the FNDs showed multi-exponential decays with intensity weighted average lifetimes <*τ*> between 5–25 ns. For an optimized gCW-STED performance, we have therefore adjusted *T_g_* = 1.5 ns and *T_g_* = 6 ns for the beads and the FNDs, respectively (Figure 3).

**Figure 4:**
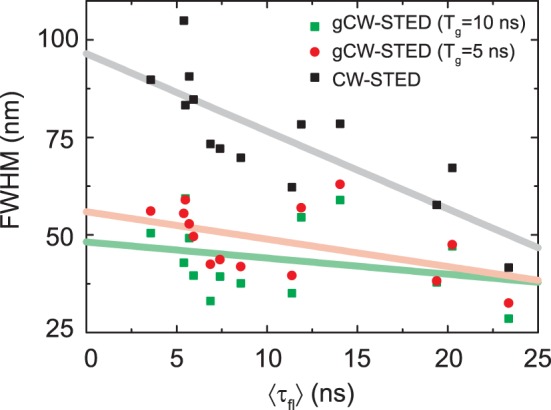
Dependence of the confinement of the E-PSF of CW-STED and gCW-STED recordings on the fluorescence lifetime. Correlative plot of values pairs of intensity-weighted average lifetime <*τ*> (*i* = 3) and FWHM of the intensity profiles through images of individual FNDs. The solid lines show linear regression fits to the experimental data with slopes of −1.99, −0.70 and −0.41 for *T_g_* = 0 ns, 5 ns and 10 ns, respectively. Imaging conditions as in Figure 3B.

On the other hand, *τ* depends very sensitively on the local molecular environment of the fluorophore and may thus vary over the sample. We have chosen the FNDs as an example to outline the implication of such variation on the performance of (g)CW-STED. As mentioned above and reported previously [33], [43], [44], the average lifetimes <*τ*> of the FNDs are heterogeneous, varying from 5 to up to 25 ns (see also Supplementary Figure S3). Applying a constant STED intensity and a fixed gate *T_g_*, the heterogeneity in lifetimes resulted in a variation of the E-PSF from one FND to the next. Figure 4 shows the correlation between the lifetime and the FWHM of the E-PSF determined from the gCW-STED images of different single isolated FNDs. Without gating, i.e., for CW-STED, shorter lifetimes result in a less confined E-PSF. Time gating reduces this dependence: While the FWHM of the E-PSFs of the pure CW-STED recordings decreases by a factor of two for lifetime values from 5 to 25 ns, this factor is only 1.2 for the gCW-STED images with *T_g_* = 5 ns and even less for *T_g_* = 10 ns. This directly follows from Equation (12), since the dependence on *τ* becomes very weak for the gated gCW-STED recordings with *T_g_*>*τ*. Consequently, at the expense of loosing signal from short-lifetime emitters, gating reduces bias due to lifetime heterogeneities of the recorded fluorophores.

**Figure 5:**
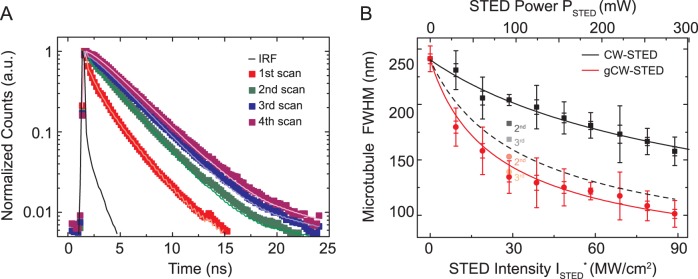
Impact of self-quenching in gCW-STED nanoscopy. (A) Fluorescence lifetime decays from consecutive TCSPC recordings of microtubules of fixed PtK2 cells immunolabeled with the dye ATTO647N. Imaging conditions as in Figure 3C. The data can be described by a two-exponential decay for the first two scans (1st: *τ*_1_ = 2.3 ns (30%) and *τ*_2_ = 0.3 ns (70%) with <*τ*> = 1.9 ns; and 2nd: *τ*_1_ = 2.8 ns (60%) and *τ*_2_ = 0.35 ns (40%) with <*τ*> = 2.6 ns) and a mono-exponential decay for the 3^rd^ and 4^th^ scan (3.1 ns and 3.3 ns). IRF: instrumental response function. (B) FWHM values determined from intensity line profiles across CW-STED (black and grey) and gCW-STED (red) images of ATTO647N-immunolabeled single microtubules for different STED intensities and for the different image scan numbers (2^nd^ and 3^rd^). Solid lines: Fit of √(8ln(2)×(FWHM^2^_gCW_+FWHM^2^_tub_) to the CW-STED (black) and gCW-STED (red) data (1st scan) (FWHM^2^_gCW_ given by Equation 12, FWHM^2^_tub_ = 50 nm the estimated size of the antibody-labelled microtubule, *d*_c = _235 nm, *a* = 3.22·10^−3^ nm^−1^, and τ = 3.15 ns, *T*_g_ *= *0 ns (CW-STED), *T*_g_ *= *1 ns (gCW-STED), resulting in I_s_^*^_ = _37.8 MW/cm^2^ (CW-STED) and 11.7 MW/cm^2^ (gCW-STED)). Grey dotted line: FWHM values expected for CW-STED with I_s_^*^_ = _11.7 MW/cm^2^ and τ = 3.15 ns.

We also observed a change in fluorescence lifetime for the dye ATTO647N when applied for immunolabeling. Attachment of several Atto647N dyes to an antibody shortens the average fluorescence lifetime due to concentration-quenching of these labels [45]. Figure 5A shows representative fluorescence lifetime decays determined from our ATTO647N-antibody labelled microtubule (compare Figure 3C). During the first image scan, the lifetime of ATTO647N (averaged over all pixels) can only be described by a two-exponential decay with an intensity-weighted lifetime of <*τ*> = 1.9 ns and a significant (70%) contribution of a short lifetime component of *τ* = 0.3 ns. This is significantly different from the lifetime of ATTO647N in aqueous solution, where its excited state decays mono-exponentially with a lifetime of 3.4 ns. Interestingly, the lifetime of ATTO647N in the immunolabeled samples increased for subsequent image scans to a final value of *τ* = 3.3 ns with a more-and-more mono-exponential decay. Most probably, the continuous photobleaching of an increasing number of the dye molecules with each scan, not only reduces the total signal but also the concentration-quenching. While this change influences consecutive CW-STED recordings, gCW-STED recordings are less affected as illustrated in Figure 5B. The effective resolution (expressed as the FWHM values determined from intensity line profiles across single microtubules) was rather low for the initial CW-STED recordings and increased with successive image scans. In contrast, there is hardly any influence on the image scan number for gCW-STED. This directly follows from Equation (12), where for CW-STED (*T_g_* = 0) an increase in *τ* immediately translates into a reduction of FWHM and thus an improvement of the effective resolution; this change in FWHM or effective resolution with *τ* is significantly reduced for *T_g_* >0. Notably, the effective resolution of the CW-STED recordings for increasing imaging runs approaches the effective resolution expected from theory (grey-dotted line).

**Figure 6:**
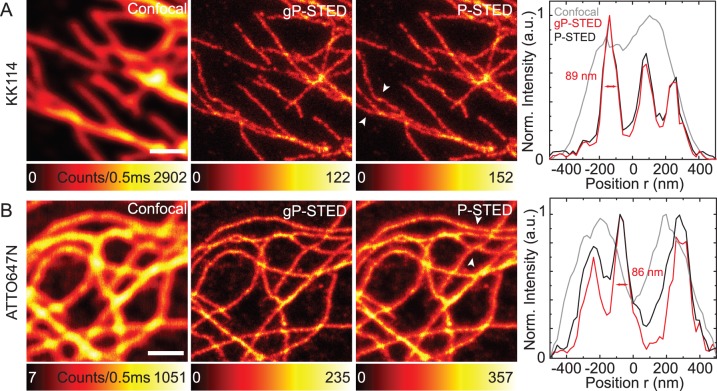
Scanning gP-STED nanoscopy images. Scanning images of microtubule of fixed PtK2 cells immunolabeled with the dye KK114 (A) and ATTO647N (B): confocal (left), time-gated gP-STED (middle left) and non-gated P-STED (middle right) images, and normalized intensity profiles along the arrows marked in the P-STED images. Excitation: 635 nm, *f* = 76 Mhz, *P_exc_*_ = _5 µW; STED: 760 nm, *f* = 76 Mhz, *P_STED_*_ = _45 mW (a), 70 mW (b); gated detection: *T_g_* = 500 ps. Pixel size: 20 nm. Scale bars: 1 µm.

We note that there are other ways to minimize concentration-quenching, for example by optimizing/minimizing the labelling degree (which may however be weighed against a reduction of the overall brightness of the labelled structures) or by using dye-labels that show less self-quenching (as for the dye KK114 shown in Figure 6A).

### gP-STED: Gating with Pulsed STED Lasers

For STED recordings using pulsed excitation and pulsed STED light (gP-STED), theory predicts an increase in image contrast for gated detection with a delay time that is smaller or equal to the pulse width of the STED laser, *T_g_*≤*T_STED_*, but not further on, *T_g_*>*T_STED_* (Figure 1). Therefore, it has been well accepted that in a pulsed STED scheme, the photons should ideally be detected right after the STED pulse [24], [29], [30]. However, in most cases the pulse width of the STED laser (≈100–300 ps) is shorter than the excited-state lifetime *τ* of the fluorescent markers (≈1–4 ns). Consequently, time-gated detection should thus hardly improve the image contrast in the pulsed STED modality. This is shown in the P-STED images of Figure 6A, where we imaged microtubule of fixed PtK2 cells, which were labeled with the dye KK114 [32]. The lifetime of KK114 in this sample was rather long (*τ* ∼ 3 ns), and the fluorescence emission of KK114 on the antibody was not quenched. Consequently, with a pulse width *T_STED_*<300 ps the gated and non-gated images are non-discernable. However, this is different for lifetimes *τ* that are in the range of the pulse width *T_STED_* as for our samples that were immunolabeled with the dye ATTO647N, which showed a strong component with a short fluorescence lifetimes *τ* = 0.3 ns ≈*T_STED_* (compare Figure 5). As Figure 6B depicts and as expected from theory, gating in this case (and only in such cases) leads to an improvement in contrast. Note that in a well implemented gP-STED scheme, the influence of the fluorescence lifetime on the spatial resolution does not exist, as becomes obvious from Equation (10). Therefore, lifetime heterogeneities ideally do not influence gP-STED resolving power.

## Discussion and Conclusions

The efficiency of inhibiting the spontaneous emission of a fluorescent marker increases with the duration of the STED beam action, as long as this duration is within the range of the lifetime of the fluorescent state or shorter. Time-gated detection uses this fact to improve the effective resolution of STED nanoscopy. This improvement is most significant for the modality using the combination of pulsed excitation with CW-STED lasers, while for an all-pulsed laser implementation (P-STED) the improvement becomes small if the STED pulse duration is much shorter than the excited-state lifetime of the fluorescent marker. We have shown that in some experimental cases, where the excited-state lifetime is shortened by inter or intra-molecular quenching, time-gated detection also improves the contrast of P-STED imaging.

STED nanoscopy, as all nanoscopy (superresolution) techniques, overcomes the diffraction resolution limit by shuffling the fluorescent marker between two distinguishable states. Thereby, any inhomogeneity of the transfer properties across the sample can lead to a variation in the imaging performance for all superresolution techniques. In the case of STED, a spatial variation of the fluorophore’s excited state lifetime can generate variations in the effective resolution of the imaging modality. Time-gated detection largely reduces this bias for CW-STED, and it removes it completely for P-STED implementations. A downside of this solution is that a notable part of the signal of short-lifetime fluorophores is reduced and may be even lost. As already discussed the loss of signal is acceptable as long as the feature or molecule is identifiable and separable from its neighbours, i.e SNR and SBR do not degrade drastically.

Given the required pulsed lasers and wavelengths are available, the use of entirely pulsed (P-STED) systems currently still remain the methods of choice, especially if the fluorophore shows little photobleaching scaling with (higher orders of) the applied STED beam intensity. Yet, time-gated detection can alleviate the performance difference between the P- and CW- STED modalities remarkably well. We showed that in combination with gated detection, the moderate instantaneous light intensities realized with CW-STED sources can in many cases provide similar resolution as pulsed systems. The main practical limitation of the gCW-STED implementation is the inherent loss of ‘good’ signal stemming from the location of the zero and the concomitant compromise in signal-to-noise and signal-to-background ratios. It should be noted that applying the gate does not increase transmission of high spatial frequencies (which are already present in the conventional CW-STED image) but rather acts as a spatial frequency filter which is able to selectively reduce the low spatial frequency contribution, thus boosting the relative strength of high resolution signal. Even so, for customary imaging parameters, time-gated detection greatly improves the effective resolution in CW-STED imaging, and helps to reveal finer details in the sample.

In our gCW-STED and gP-STED implementation the images were realized off-line using the TCSPC image measurement and selecting only those photons recorded after a time-delay *T_g_* from the excitation pulse. However, fast electronic detection gates can be realized to obtain real-time images [32]. Simply disregarding the photons that arrive outside the gate is somewhat wasteful, because they too carry spatial information about the sample. We therefore anticipate a further improvement from combining TCSPC measurements with new methods of deconvolution that take into account the time-dependent E-PSF of a CW-STED microscope [46], [47].

## Supporting Information

Figure S1 **Scanning gCW-STED nanoscopy with negligible uncorrelated backgrounds.** gCW-STED nanoscopy of ∼40 nm fluorescent crimson beads (compare Fig. 3A). (Upper panel) gCW-STED images with *T_g_* = 1.5 ns (PSNR ∼10), 3 ns (PSNR ∼8), 6 ns (PSNR ∼4) and 9 ns (PSNR ∼2); (Lower panel) normalized an un-normalized intensity profiles along the arrows marked in the upper panel.
(EPS)

Figure S2 **Scanning gCW-STED nanoscopy with substantial uncorrelated backgrounds.** gCW-STED nanoscopy of ∼35 nm FNDs (compare Fig. 3B). (Upper panel) gCW-STED images with *T_g_* = 1.5 ns (PSNR ∼16, PSBR ∼13), 3 ns (PSNR ∼8, PSBR ∼5), 6 ns (PSNR ∼5, PSBR ∼3) and 9 ns (PSNR ∼4, PSBR ∼2); (Lower panel) normalized an un-normalized intensity profiles along the arrows marked in the upper panel.
(EPS)

Figure S3 **Image heterogeneities of the 35-nm nanodiamonds.** Correlative plot of value pairs of luminescence lifetime 〈*τ*〉 and peak signal-to-noise ratio (PSNR) (A) and full-width at half-maximum (FWHM) and PSNR (B) as determined from 14 single FNDs of the CW-STED (black) and gCW-STED recordings with Tg = 5 ns (red) and 10 ns (blue). The large variations but on the other hand the non-correlative characteristic of the value pairs demonstrates differences in the composition of each nanodiamond with respect to number of Nitrogen vacancy (NV) centers and their charges or distances to the particle surface, or due to surface in-homogeneities and contaminations.
(EPS)

Movie S1 **gCW-STED image of fluorescent beads for increasing time-delay** ***T*** **_g._** Same description as Figure 3A. Each frame is normalized to its maximum and represents a different time-delay *T*_g_ (upper left).
(ZIP)

Movie S2 **gCW-STED image of fluorescent nano-diamonds for increasing time-delay** ***T*** **_g._** Same description as Fig. 3(B). Each frame is normalized to its maximum and represents a different time-delay *T*_g_ (upper left).
(ZIP)

Movie S3 **gCW-STED image of ATTO-647N-immunolabelled microtubules for increasing time-delay** ***T*** **_g._** Same description as Fig. 3(C). Each frame is normalized to its maximum and represents a different time-delay *T*_g_ (upper left).
(ZIP)

Text S1 **Full-width at half-maximum of the gCW-STED point spread function.**
(DOC)
