# Supplementary material for: STED Nanoscopy with Time-Gated Detection: Theoretical and Experimental Aspects
Source: PLoS One. 2013 Jan 18;8(1):e54421. doi: 10.1371/journal.pone.0054421 (PMC3548795; doi:10.1371/journal.pone.0054421)
Supplement: Text S1 — Full-width at half-maximum of the gCW-STED point spread function. (DOC) [file pone.0054421.s007.doc]

**Full-width at half-maximum of the gCW-STED point spread function**

Without approximating the Lorentzian function in Eq. (9) with a Gaussian function, the FWHM of the gCW-STED modality is given by:

$FWHM_{gCW}=d_{c}\sqrt{\left(W\left(2xe^{x}\right)-x\right)/\ln2}/\sqrt{1+d_{c}^{2}a^{2} \varsigma^{*}T_{g}/\left(\tau\ln2\right)}$, (1)

with *x* = ln2/(*a*^2^ζ^*^*d*^2^) + *T*_g_/τ and *W* being the Lambert W-function. For small *x* the first square-root is approximated by ~(*x*ln2)^1/2^ and for large x it tends to unity. Thus FWHM_gCW_ approaches FWHM_gP_ for long gate delays *T*_g_ and d_c_/*a*√ζ^*^ for short delays *T*_g_ and large saturation factors, i.e. large STED intensities.
